# Introduction

**DOI:** 10.1155/EDR.2003.133

**Published:** 2003


*Experimental Diab. Res.*, 4:133, 2003
Copyright © Taylor and Francis Inc.
ISSN: 1543-8600 print / 1543-8619 online
DOI: 10.1080/15438600390261426

### Introduction

The 9th International Workshop on Lessons from Animal Diabetes was held June 17–21, 2003 in Bar Harbor, Maine, USA. The title of the Workshop was "New Genetic and Metabolic Insights into Animal Models of Diabetes" and it comprised many data on new models of diabetes discovered and defined since the last meeting. The meeting was prepared and led by Edward H. Leiter from the Jackson Laboratories in Bar Harbor. There were over 200 participants and the conference venue facilitated good interaction between participants and the effective delivery of the material.

The Workshop was divided into two concurrent tracks: Models of diabetes type 1 and type 2. Each session started with a plenary lecture which were given by John Mordes on Autoimmune Diabetes in the Rat; Richard Flavell on Genetic Manipulation of Immune and Autoimmune Response; Hiroshi Ikegami on Genetics of Type 1 and Type 2 Diabetes in Inbred Strains Derived from the Same Outbred Colony; and Leonard Shultz on Humanized Mouse Models for Transplant Research. An important feature was the most interesting and novel Albert Renold Memorial lecture given by Ronald C Kahn, entitled "Unraveling Insulin Action and the Pathogenesis of Diabetes Through the Use of Knockout Mice." This lecture appears in this issue together with the abstracts of the communications and posters presented in the Workshop.

The final feature of the meeting was a gala dinner at which Douglas Coleman gave an exciting report on his lifetime work at the Jackson Laboratory which included the specific genetic and metabolic deficiencies of the *ob*/*ob* and *db*/*db* mutant mice, which led to the discovery of leptin.

It was the feeling of all participants that the studies with animal models are presenting new fields of diabetes research and contribute significantly to the understanding of pathogenesis of human diabetes in new and hitherto used models. This represents an important contribution to the understanding of pathogenesis of human diabetes and an incentive to the development of new therapeutic modalities.

We wish to thank all the participants for their important presentations and lively discussions and the organizers for creating a most pleasant ambience for the meeting including a visit to the Jackson Laboratories and the Bar Harbor surroundings. The program committee included: Edward H. Leiter, David Serreze, Hiroshi Ikegami and Eleazar Shafrir. In Bar Harbor it was proposed and accepted to create a Society for Experimental Diabetes Research, with an annual membership fee of US$70. This fee will include subscription to this Journal and substantially reduced registration fees to future LAD Workshops. Further invitations to join the Society and information on its function, its by-laws and proposal for activities will be distributed in the near future.

